# SwissTecLive: effectiveness and safety of dimethyl fumarate in the treatment of RRMS in the Swiss clinical practice setting

**DOI:** 10.1016/j.heliyon.2020.e05819

**Published:** 2020-12-23

**Authors:** Chiara Zecca, Adam Czaplinski, Christophe Henny, Liliane Petrini, Andreas Beeler, Claudio Gobbi

**Affiliations:** a Neurocenter of Southern Switzerland, Ospedale Regionale di Lugano, Lugano, Switzerland; b Faculty of Biomedical Sciences, Università della Svizzera Italiana, Lugano, Switzerland; c Neurozentrum Bellevue, Theaterstrasse 8, 8001 Zurich, Switzerland; d Clinique de La Source, Avenue Bergières 2, 1004 Lausanne, Switzerland; e Biogen Switzerland AG, Neuhofstrasse 30, 6340 Baar, Switzerland

**Keywords:** Dimethyl fumarate, Multiple sclerosis, Patient support, Post-marketing survey, Switzerland

## Abstract

**Background:**

Delayed-released dimethyl fumarate (DMF) is an oral disease-modifying therapy (DMT) approved for treating patients with multiple sclerosis (MS). This post-marketing study aimed at collecting real-world data on the safety, effectiveness, and tolerability of DMF in patients with relapsing remitting multiple sclerosis (RRMS).

**Methods:**

1-year post-marketing survey of patients prescribed DMF followed-up quarterly in hospital setting and private neurological practices in Switzerland from January 2015 to January 2018. Data on relapses, Expanded disability status scale (EDSS) score change, safety, tolerability, treatment adherence as judged by the treating neurologist and satisfaction were collected. Patients could refer to a patient support program.

**Results:**

Of the 158 patients, 67 (42.4%) were treatment naïve, 91 (57.6%) switched from a prior MS DMT to DMF, 131 (82.9%) were treatment adherent, 108 (68.4%) used the support program, and 45 (28.5%) discontinued the therapy. Insufficient tolerability and insufficient effectiveness were the main reasons for discontinuation. 134 (84.8%) patients remained relapse free, 97 (61.4%) had stable or decreased EDSS score after 12 months. 74 (46.8%) patients reported adverse events; of these, 28 (17.7%) discontinued DMF treatment. Physicians and patients rated treatment satisfaction similarly (median score 8.0 of 10).

**Conclusions:**

The results obtained from this real-world observation are consistent with the efficacy and safety findings reported in pivotal and larger observational trials evaluating DMF treatment. Most side effects were experienced early after therapy initiation reflecting the timing of therapy discontinuation.

## 1 Introduction

Multiple sclerosis (MS) is a chronic inflammatory disease of the central nervous system (Reich et al., 2018). Its prevalence in Switzerland was estimated to range between 0.15% to 0.19% from 2011 to 2015 with increasing tendency (Blozik et al., 2017). In the past years, new drugs with refined mode of actions or approved drugs with new dosage forms entered the market (Coyle and Markowitz, 2018; Montalban et al., 2018; Rae-Grant et al., 2018a, Rae-Grant et al., 2018b). To benefit from the vast armamentarium of MS drugs, good access to neurological care, effective risk communication, and patients' awareness of the importance to be treatment adherent and persistent are crucial (Costa et al., 2015; Patti, 2010; WHO report, 2003).

Possible strategies to enhance adherence and persistence to MS therapies are advances in delivery technology, improved patient education and support, alternative administration routes (Patti, 2010), and a specific patient education and support programs (Ganguli et al., 2016; Groeneweg et al., 2018; Roche et al., 2014; Stockl et al., 2010). The availability of orally administered drugs is anticipated to significantly improve long-term treatment adherence (Patti, 2010). One of four oral treatments for relapsing-remitting multiple sclerosis (RRMS) available on the Swiss market is delayed-released dimethyl fumarate (DMF). In phase 3 pivotal trials DMF compared to placebo significantly reduced the relapse rate (Fox et al., 2012; Gold et al., 2012) and disability progression (Gold et al., 2012) n patients with RRMS. A reduced annual relapse rate in patients treated with DMF was confirmed also in real world settings (Giles et al., 2019; Mallucci et al., 2018). In the clinical trials the number of patients receiving DMF who experienced flushing events and gastrointestinal (GI) events comprising diarrhea, nausea, upper abdominal pain, abdominal pain, vomiting, and gastritis was highest in the first month of treatment and declined in the second month (Fox et al., 2012; Gold et al., 2012). In real-world settings, adverse events related to DMF tolerability are more likely to occur during the initial phase of DMF treatment and are the main reasons for treatment discontinuation (Begus-Nahrmann et al., 2015, Begus-Nahrmann et al., 2016; Sammarco et al., 2014). After launch in Switzerland in 2014, a post-marketing survey collected data on real-world effectiveness and tolerability of DMF in Swiss RRMS patients who were either treatment naïve or switching from any first DMT to DMF and who were followed-up according to routine clinical practice.

We present the data of this post-marketing survey.

## 2 Methods

### 2.1 Setting

This was a 1-year, post-marketing survey of DMF use prescribed to RRMS patients in Switzerland initiated by Biogen Switzerland AG conducted from January 2015 until January 2018.

Specialized private neurological practices and hospital ambulatories in Switzerland experienced with DMF were contacted 6 months after launch. Interested neurologists could register on-line to participate. Eligible were consenting adult patients with relapsing remitting multiple sclerosis having been prescribed DMF and being therapy naïve or with one prior disease modifying therapy. Treatment with DMF was started within 2 weeks of Visit 1, and the dose of DMF was progressively increased as per standard clinical practice (120 mg/day for a week, then 240 mg/day for a week, then 360 mg/day for a week, then 480 mg/day in two oral administrations). Pre-specified clinical data were recorded at baseline (Visit 1) and every 3 months thereafter (visits 2, 3, 4 and 5), for a total follow-up of 12 months. Data were captured electronically in the database set-up for this survey and hosted by Ogilvy.

At Visit 1 information on demographic data, MS history, previous therapy, number of relapses during the previous 12 months, and the last EDSS score were collected. The patients were informed about support options. At Visits 2 through Visit 5, treatment satisfaction, rated on a 10-point scale (1 not at all satisfied, 10 very satisfied), clinical parameters such as occurrence of relapses (isolated fatigue or urinary symptoms as well as any neurological worsening during fever were not considered relapses according to current clinical practice) (Thompson et al., 2018), adverse events, therapy continuation, and adherence were recorded. Also at Visit 5 each patients' expanded disability status scale (EDSS) score (Kurtzke, 1983) was captured. This post-marketing setting did not allow to perform drug accountability for treatment adherence and discontinuation as in a clinical trial. Therefore, the neurologists judged treatment adherence during the consultations and together with the patient decided on DMF treatment continuation or discontinuation.

To enhance treatment compliance even in case of initial tolerability issues patients were informed and some were registered to the support program TecCare provided by MediService AG on behalf of 10.13039/100005614Biogen Switzerland AG. The services provided in this support program contained a hotline to receive immediate counseling for emerging queries, to ask for monthly calls during the first 6 months on therapy, or to arrange for a home visit by a specialized MS nurse.

### 2.2 Statistical methods

All numerical outcomes were analyzed descriptively computing means, minimal and maximal values. Categorical variables were analyzed presenting the absolute and relative observed frequencies. Several clinical variables were tested for association with time to relapse, adverse events and DMF discontinuation using multivariate Cox regression models. P values less than 0.05 were considered statistically significant.

### 2.3 Ethics

The patients signed an informed consent form to allow collection and transfer of their clinical practice data related to the treatment with DMF.

No approval by the Swiss ethics committees was required since this post marketing survey was not a research project subject to the Human Research Act. Nevertheless, all national ethic committees (Zurich, North-West and central Switzerland, Bern, Geneva, St. Gallen, Thurgau, Tessin/Ticino, Waadt/Vaud and Wallis/Valais) were notified about this survey. They received all relevant documents including the informed consent form, the data collection form as well as the information sheets on the support program handed out to the doctors and the patients.

## 3 Results

### 3.1 Setting and patient disposition

Twenty-seven physicians from private practices and hospital ambulatories participated and observed 158 patients from January 2015 to January 2018. Average (range) follow up was 56 (41–129) weeks. The mean number of weeks elapsed between visits ranged between 13.4 and 14.3. One hundred-nineteen patients (75.3%) were female. The average age (range) was 41 ± 9 (18–73) years. Patients had a mean (range) disease duration of 5.1 ± 6.4 (0–37) years, 67 (42.4%) were treatment naïve, 91 (57.6%) switched from a prior MS DMT to DMF. Sixty (38.0%) patients were previously treated with interferon β1, 7.6% with glatiramer acetate, 7.0% with fingolimod, 3.2% with natalizumab, 1.3% with teriflunomid, 0.6% with another fumarate. In the year before therapy start, the mean (range) number of relapses was 0.7 ± 0.7 (0–3) and the last mean (range) EDSS score was 2.0 ± 1.3 (0–6.5) assessed 2.4 ± 6.8 weeks before DMF start (Table 1).

**Table 1:** Characteristics of patients.

Parameters	Values
Number of patients, N (%)	158 (100)
Observation time, weeks, mean (range)	56 (41–129)
Male/Female, N (%)	39 (24.7)/119 (75.3)
Age at visit 1, mean, years (SD; range)	41 (9; 18–73)
Disease duration since MS diagnosis, mean, years (SD; range)	5.1 (6.4; 0–37)
Treatment-naïve patients	67 (42.2%)
First switch from another MS therapy to DMF	91 (57.6%)
Previous basic treatments of RRMS, N (%)	
Interferon-β	60 (38.0%)
Glatiramer acetate	12 (7.6%)
Fingolimod	11 (7.0%)
Natalizumab	5 (3.2%)
Teriflunomide	2 (1.3%)
Alemtuzumab	0 (0.0%)
other fumarate	1 (0.6%)
Number of MS relapses in the last 12 months, mean (SD; range)	0.7 (0.7; 0–3)
Last EDSS score – grade, mean (SD; range)	2.0 (1.3; 0–6.5)

Overall, 45 (28.5%) patients discontinued DMF treatment: 16 (10.1%) at V2, 11 (7.0%) at V3, 8 (5.1%) at V4, and 10 (6.3%) at V5. The median time to treatment discontinuation was 26 weeks.

In total, 108 (68.4%) patients referred to the support program. One-hundred thirty-one (82.9%) patients were judged to be treatment adherent, whereas 55 (34.8%) patients were treatment naïve, 24 (15.2%) discontinued the therapy, and 82 (51.9%) used the support program. Twenty-seven (17.1%) patients were non-adherent, with 12 (7.6%) being treatment naïve, 21 (13.3%) who discontinued the therapy, and 26 (16.5%) who used the support program (Table 2).

**Table 2:** Disposition of patients.

Parameter	Number of patients∗ N = 158
	N (%)
Therapy discontinuation	45 (28.5)
at V2	16 (10.1)
at V3	11 (7.0)
at V4	8 (5.1)
at V5	10 (6.3)
for insufficient tolerability [V2, V3, V4, V5]	21 (13.3) [9, 6, 3, 3]
for insufficient effectiveness [V2, V3, V4, V5]	11 (7.0) ([0, 1, 3, 7]
for pregnancy and desire to have children	5 (3.2)
Other	12 (7.6)
Use of support	108 (68.4)
Treatment adherent	131 (82.9)
Treatment naïve	55 (34.8)
First switch	76 (48.1)
Therapy discontinuation	24 (15.2)
Using support	82 (51.9)
Not treatment adherent	27 (17.1)
Treatment naïve	12 (7.6)
First switch	15 (9.5)
Treatment discontinuation	21 (13.3)
Using support	26 (16.5)

### 3.2 Efficacy

During the observation period, 134 (84.8%) patients remained relapse-free, 17 (10.8%) experienced 1 relapse, and 7 (4.4%) experienced ≥2 relapses when treated with DMF. The number of patients with relapses was similar in the treatment naïve group and the group with prior DMT therapy (p = 0.65, global Fisher-test). The mean number of relapses experienced during the whole observation period was 0.196 ± 0.498 (n = 24). The mean EDSS score change from V1 to V5 was -0.175 ± 0.814 (n = 120). Fifty-six (35.4%) patients had no change of the EDSS score, 41 (25.9%) had decreased, and 23 (14.6%) increased EDSS scores at V5 after 12 months. The number of patients with an EDSS score change was similar between treatment naïve patients and those with prior DMT (p = 0.31, global Fisher-test) (Table 3).

**Table 3:** Number of relapses and EDSS score changes in treatment naïve patients and patients with prior disease modifying treatment.

	Relapses				EDSS change			
	0	1	2	p-value†	Decrease	No change	Increase	p-value†
All patients	134	17	7		41	56	23	
Treatment naïve	55	9	3	p = 0.65	21	20	10	p = 0.31
First switch to DMF	79	8	4		20	36	13	

† Global Fisher Test.

### 3.3 Safety and tolerability

Seventy-four (46.8%) patients reported adverse events (AE) leading to treatment discontinuation in 28 (17.7%) patients. Eleven (7.0%) patients discontinued DMF treatment at V2, 6 (3.8%) at V3, 5 (3.2%) at V4, and 6 (3.8%) at V5. 16 (10.1%) patients discontinued DMF at the same visit when the AE was reported. Fifty-two (32.9%) patients with AEs used the support program, but only 13 (8.2%) before the adverse event occurred.

Tolerability for GI events or flushing improved at least once between two visits in 44 (27.8%) patients, in 31 (19.6%) already after the first 14 weeks of treatment with DMF. Thirty (19.0%) patients had no improved tolerability for GI events or flushing during the whole observation period (Table 4).

**Table 4:** Patients with adverse events.

Parameter	Number of patients N = 158
	N (%)
≥1 adverse event	74 (46.8)
≥1 adverse event and therapy completed	46 (29.1)
≥1 adverse event and therapy discontinued	28 (17.7)
at V2	11 (7.0)
at V3	6 (3.8)
at V4	5 (3.2)
at V5	6 (3.8)
at the same visit as AE reported [V2, V3, V4, V5]	16 (10.1) [11, 2, 3, 0]
Use of support	52 (32.9)
before first adverse event	13 (8.2)
Tolerability increased for gastrointestinal events or flushing V2–V5	44 (27.8)
at V2	31 (19.6)
at V3	12 (7.6)
at V4	11 (7.0)
at V5	8 (5.1)
Tolerability not increased for gastrointestinal events or flushing V2–V5	30 (19.0)
at V2	24 (15.2)
at V3	15 (9.5)
at V4	12 (7.6)
at V5	8 (3.2)

### 3.4 Variables associated with occurrence of adverse events, relapses and DMF discontinuation

We used multivariate Cox regression models to test age, gender, treatment status (naïve vs with prior DMT), EDSS and being registered for TecCare program for association with time to relapse, adverse event and DMF discontinuation. No variables appeared significantly associated with risk of relapse and risk of DMF discontinuation (Table 5). Instead, female gender, a lower EDSS, treatment naïve status and being registered for TecCare program were all associated with a shorter time to adverse events (Table 5).

**Table 5:** Cox regression models testing the association of several clinical variables with time to first adverse event, time to first relapse and time to DMF discontinuation.

Predicted	Predicting	HR	95%CI	p
Time to adverse event	Gender (F)	1.78	1.00–3.16	0.049
	Age	1.02	0.99–1.04	0.204
	EDSS	0.75	0.59–0.95	0.017
	Previous treatment (naive)	2.14	1.31–3.49	0.002
	TecCare (registered)	2.34	1.24–4.40	0.008
Time to relapse	Gender (F)	0.56	0.23–1.36	0.202
	Age	0.95	0.90–0.99	0.018
	EDSS	1.36	0.92–2.01	0.123
	Previous treatment (naive)	1.47	0.60–3.58	0.397
	TecCare (registered)	1.42	0.40–5.01	0.582
Time to DMF discontinuation	Gender (F)	1.64	0.74–3.60	0.221
	Age	0.98	0.95–1.01	0.130
	EDSS	1.16	0.87–1.53	0.312
	Previous treatment (naive)	1.14	0.59–2.19	0.691
	TecCare (registered)	1.89	0.81–4.40	0.141

### 3.5 Reasons for therapy discontinuation

Reasons for DMF treatment discontinuation (multiple answers) includes insufficient tolerability (n = 21, 13.3%), insufficient effectiveness (n = 11, 7.0%), pregnancy or desire to have children (n = 5, 3.2%) and other reasons (n = 12, 7.6%) (Table 2).

### 3.6 Treatment satisfaction

**Figure 1:**
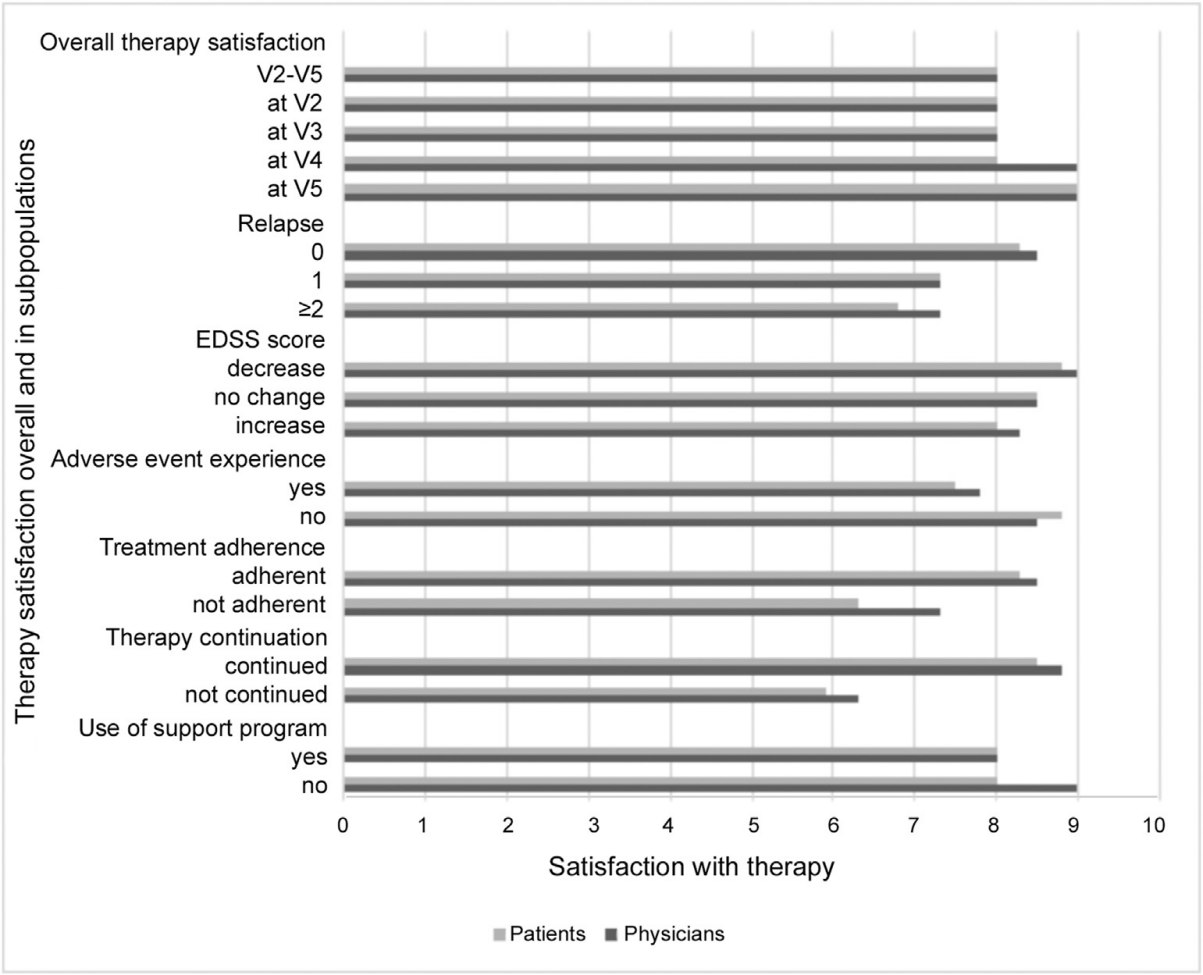
Therapy satisfaction of patients and physicians rated on a 10-point scale (1 not at all satisfied, 10 very satisfied), median values.

Physicians and patients rated treatment satisfaction similarly (physicians: median 8.0/10; patients: median 8.0/10; 95% CI physicians 4.6–10, patients 3.0–10). Median satisfaction at V5 was increased by one point (10%) (physicians 9.0, 95% 5.2–10; patients 9, 95%CI 4.5–10). Median treatment satisfaction depended on treatment success being rated lower in patients experiencing relapses, with EDSS score progression, having experienced an adverse event, and consequently in those not treatment adherent and who discontinued (Figure 1).

## 4 Discussion

According to this post-marketing survey in a small Swiss cohort starting DMF therapy, clinical practice setting data is in line with the favorable efficacy and safety of DMF reviewed in the literature (Linker and Haghikia, 2016) and emerging from post-marketing studies in real-world settings (Berger et al., 2019; Miclea et al., 2016; Mirabella et al., 2018). The majority of our patients remained relapse free and had stable or decreasing EDSS scores. The discontinuation rate of 28.5% in our population was overall comparable with those reported in Denmark (27.7%) and Italy (24%) (Sejbaek et al., 2018; Ferraro et al., 2018). This also concurs roughly with a Swedish population based cohort study, in which 34% DMF naïve patients stopped treatment within 12 months (Eriksson et al., 2018). DMF discontinuation was related to tolerability issues in 13.3% and lack of effectiveness in 7% of the patients, with similar figures also reported in other studies (Saccà et al., 2019). As to be expected, in our population most adverse events were experienced early after therapy initiation reflecting also the timing of therapy discontinuation. A similar trend was detected in a clinical practice study over 12 months comparing DMF with other oral DMTs (Hersh et al., 2016). The observed improved tolerability related to gastrointestinal events or flushing early after therapy initiation is in line with findings from the pivotal trials (Fox et al., 2012; Gold et al., 2012; Havrdova et al., 2013) and usually increases over time (Linker and Haghikia, 2016) as seen also in our cohort with 27.8% of the patients experiencing increased tolerability of gastrointestinal events. Interestingly, the risk of adverse events was especially higher among patients who were treatment naïve and were registered within the TecCare program. These findings are interesting and we speculate that being less adapted to treatments (as naïve patients) and, more importantly, being registered under a monitoring program may increase self-awareness, the likelihood of reporting adverse events and their adequate treatment.

The adherence rate to DMF in this real-world setting is in line with that reported in a retrospective study in MS patients of an integrated health system (Hao et al., 2017).

Several retrospective studies and some in real-world settings concur on the importance of counseling for DMF tolerability and GI management to enhance treatment adherence and prevent therapy discontinuation (Begus-Nahrmann et al., 2015, 2016; Min et al., 2019; Niemczyk et al., 2018; Phillips et al., 2016). Patients referred more to the support program after the experience of an adverse event.

Treatment satisfaction in our cohort was generally high.

This investigation is limited to a small number of patients in a confined geographical area benefitting from a well-developed public health care system. Treatment adherence could not be measured objectively in this clinical practice survey.

## 5 Conclusions

Real-life experience of this cohort is in line with the favorable efficacy and safety profile of DMF reviewed in the literature and confirmed by the high adherence. Experiencing an adverse event motivated the patients to refer to a support. Personalized counseling to emphasize the importance of treatment adherence and to provide management strategies especially for gastrointestinal events may encourage patients to stay on treatment, remain adherent or come to an informed decision to discontinue the therapy.

## Declarations

### Author contribution statement

C. Zecca: Conceived and designed the experiments; Analyzed and interpreted the data; Wrote the paper.

A. Czaplinski, C. Henny and C. Gobbi: Conceived and designed the experiments; Analyzed and interpreted the data.

L. Petrini: Analyzed and interpreted the data.

A. Beeler: Conceived and designed the experiments.

### Funding statement

This work was supported by 10.13039/100005614Biogen, Switzerland.

### Data availability statement

Data included in article/supplementary material/referenced in article.

### Declaration of interests statement

The authors declare the following conflict of interests: C. Zecca received honoraria for speaking, consulting fees, or research grants from Abbvie, Almirall, Biogen Idec, Celgene, Merck, Novartis, Sanofi, Teva Pharma, Roche. A. Czaplinski received speaker and consulting fees from Alimirall, Biogen, Celgene, Merck, Novartis, Roche, Sanofi, TEVA Pharma. C. Henny received honoraria for consulting fees from Biogen, Sanofi-Genzyme, Merck Serono, Novartis, Teva Pharma, Roche. A. Beeler is an employee and stockholder of Biogen. C. Gobbi received honoraria for speaking, consulting fees, or research grants from Abbvie, Almirall, Biogen Idec, Celgene, Merck, Novartis, Sanofi, Teva Pharma, Roche.

### Additional information

No additional information is available for this paper.
